# Non-Operative Management for Osteochondral Lesions of the Tibial Plafond Results in Minor Improvements of Patient-Reported Outcomes: A 2-Year Prospective Follow-Up Study

**DOI:** 10.1177/19476035251376180

**Published:** 2025-09-27

**Authors:** Quinten G.H. Rikken, Jari Dahmen, Sjoerd A.S. Stufkens, Gino M.M.J. Kerkhoffs

**Affiliations:** 1 Department of Orthopedic Surgery, Amsterdam Movement Sciences, Amsterdam UMC, Location AMC, University of Amsterdam, Amsterdam, The Netherlands; 2 Academic Center for Evidence Based Sports Medicine (ACES), Amsterdam UMC, Amsterdam, The Netherlands; 3 Amsterdam Collaboration for Health and Safety in Sports (ACHSS), International Olympic Committee (IOC) Research Center, Amsterdam UMC, Amsterdam, The Netherlands

**Keywords:** ankle, tibia, OLTP, cartilage, non-operative

## Abstract

**Objective:**

Osteochondral lesions of the tibial plafond (OLTP) are considered rare, and to date the treatment for these lesions has solely focused on operative management. The aim of this study was to prospectively assess the 2-year patient-reported outcomes, radiological outcomes, and adverse outcomes for the non-operative treatment of patients with a symptomatic OLTP.

**Design:**

Eighteen patients with a symptomatic OLTP who underwent non-operative treatment were prospectively assessed. The primary outcome concerned the numeric rating scale (NRS) for pain during weightbearing from baseline to 2-year follow-up. Secondarily, the patient-reported outcomes (PROMs) NRS during rest, running, and stairclimbing, as well as the Foot and Ankle Outcome Score (FAOS) and short-form-36 (SF-36) questionnaires were assessed. CT scans at median 2 years (IQR: 1.5–2) follow-up were reviewed for changes in lesion volume or signs of lesion healing. Return to sports and work rates were evaluated. The conversion to surgery rate and any complications were assessed.

**Results:**

The NRS during weightbearing improved (non-significantly) from a median of 5 (IQR: 3–7) out of 10 at baseline to 2 (IQR: 1–6) out of 10 at 2-year follow-up, *P* = 0.06. The other NRS subscales, FAOS subscales, and SF-36 did not significantly improve at final follow-up. The follow-up CT-evaluation showed that lesion volume did not change (219 [IQR: 79–890] mm^3^) compared to baseline (226 [IQR: 79–890] mm^3^), *P* = 0.2. In 10 (77%) out of 13 cases, signs of lesion filling or no change was observed. At final follow-up, 93% (13/14) of patients returned to any level of sports, 54% (7/13) of patients returned to preinjury level of sports, and 94% (15/16) of patients returned to work. No adverse events were observed, and 1 (6%) case converted to surgery.

**Conclusions:**

Non-operative management for OLTP resulted in minor improvements of patient-reported pain and functional outcomes up to 2-year follow-up. The conversion to surgery rate was 6%. Radiologically, lesion size and filling were found to remain stable at CT follow-up. Moreover, on average 9 out of 10 patients were able to participate in sport and could return to, or remain at, their preinjury work activities.

## Introduction

Osteochondral lesions of the tibial plafond (OLTP) are considered rare, with an incidence ratio of 1 OLTP to 14–24 osteochondral lesions of the talus (OLT).^1,2^ Patients with an OLTP may present after an ankle trauma, such as a sprain or fracture, with complaints of (deep) ankle pain during weightbearing, joint swelling, and/or locking.^3,4^

To date, the treatment of OLTP is largely based on the rationale and algorithm for talar lesions, and primarily consists of operative treatment options.^5^ Recent systematic reviews found that the outcomes of these operative treatments can be considered moderate to good, but a severe limitation of the current literature is its low-quality evidence.^5,6^ It is known from the OLT literature that up-to 45% of patients show satisfactory clinical outcomes following non-operative treatment, which recedes the need for surgical treatment. Moreover, surgical treatment entails inherent surgical risks, and possibly higher costs and a longer time-off from work and sports.^7^ Even though its use is considered standard practice before commencing with operative treatment in cartilage lesions of the ankle, no literature is currently available on the non-operative management for OLTP.^5,8,9^ Therefore, no evidence-based (shared-decision) discussion can be held between patient and physician on the safety and efficacy of non-operative treatment for OLTP.

The present study, therefore, primarily aimed to prospectively assess the 2-year patient-reported outcomes for the non-operative treatment of patients with a symptomatic OLTP. Secondarily, the study assessed radiological outcomes, return to sport- and work rates, as well as adverse events and the conversion to surgery rate These results can aid in patient counseling and expectation management, while providing physicians with evidence for patient-specific treatment guidance.

## Materials and Methods

This study is a prospective, single-center, case-series with 2-year follow-up. Ethical approval for this study was obtained from the local Medical Ethics Committee at Amsterdam UMC, location AMC (reference number W14_237#14.17.0288).

### Patient Selection

All patients presenting with a symptomatic OLTP at our institution between November 2019 and July 2022 were screened for eligibility. A symptomatic OLTP was defined as a radiologically confirmed OLTP with deep ankle pain (during or after weightbearing) arising from the lesion with or without joint swelling or mechanical symptoms (such as locking or catching), and corroborative findings on physical examination (such as palpation pain of the lesion). The inclusion- and exclusion criteria for participation in the study are listed in Suppl. Table 1. Patients with an OLTP and coexisting talar lesion (OLT) were eligible for inclusion. Our institution is a tertiary academic referral hospital which is recognized as an expert center in the diagnosis and treatment of cartilage lesions of the foot and ankle.

### Non-Operative Treatment

Non-operative treatment consisted of, or the combination of, the following treatments during the follow-up period: supervised neglect, insoles or shoe modifications, physical therapy, weight-loss recommendations, or intra-articular injection with hyaluronic acid or corticosteroids (2 injections with a 2-week interval). The choice for a specific treatment was made on an individual basis, in a shared decision-making process, and was thus not standardized.

### Data Collection

Baseline demographic and treatment information were extracted from the hospital electronic patient records. Baseline demographics included sex, age, body mass index (BMI), injury circumstances, laterality, hindfoot alignment, and prior foot or ankle surgery. Treatment characteristics were primary or non-primary (i.e., failed previous surgical treatment) lesion type, any concomitant diagnosis at initial clinical evaluation, and the specific non-operative treatments utilized as previously described.

### Patient-Reported Outcome Measures

All patient-reported outcome measures (PROMs) were collected through the online CASTOR®-portal and prospectively collected by a researcher not responsible of clinical care. The PROMs that were collected concerned the numeric rating scale (NRS) of pain (during rest, during walking, during running, and during stairclimbing), the foot and ankle outcomes score (FAOS), and the Short Form-36 (SF-36). The NRS of pain is a Likert pain scale that ranges from 0 (no pain) to 10 (worst imaginable pain). The FAOS measures from 0 (lowest) to 100 (highest) and consists of 42 questions distributed among 5 subscales: symptoms, pain, activities of daily living, sport, and quality of life. The SF-36 is a general-health questionnaire with 2 subscales, the physical component scale (PCS) and mental component scale (MCS).

At baseline, the type of sport and athletic level (i.e., amateur, competitive, or professional) were recorded. At 2-years follow-up a patient-reported sports evaluation consisted of the return to sports (RTS) rate in percentages and RTS time in weeks, type of sport, and level of activities. Return to sports level was defined as according to Ardern *et al*.^10^ In a similar fashion, the baseline and 2-year postoperative patient-reported work activities and return to work time were collected.

### Radiological Assessment

Baseline CT scans were available for all patients and were assessed by 2 independent measurers (**Q.R.** and **J.D.**) for lesion characteristics. The baseline assessment consisted of lesion size (anterior-posterior direction, medial-lateral direction, and depth), lesion location according to a 9-grid scheme,^2^ the presence of cysts, and lesion morphology according to a general morphological classification for OLT.^11^

CT scans at final clinical follow-up were assessed by 2 independent measurers (**Q.R.** and **J.D.**). The assessment included lesion size measurements and signs of lesion healing (subjective increased lesion filling compared to baseline) or deterioration (subjective increased cyst formation).

### Statistical Analysis

A sample size calculation for the primary outcome, the NRS during walking from preoperatively to 2 years postoperatively, indicated that a minimum of 16 ankles were needed to detect a difference in means of 2.0 out of 10, assuming a standard deviation of 2.5 using a Wilcoxon signed-rank test with a 2-sided 0.05 significance level and 80% power (nQuery advisor 7.0, Statistical Solutions Ltd., Boston, MA). A difference of 2 points was chosen as this corresponds to a “much better” improvement in pain.^12,13^ To correct for a potential loss to follow-up of 10% the required minimum sample size for the present study was 18 cases.

Statistical analysis was performed using Stata 17 (StataCorp LP, College Station, TX). A 2-sided level of *P* < .05 was considered significant. Data normality was assessed using a Shapiro-Wilk test. Continuous baseline variables distributed normally were reported as means with standard deviations and as median with interquartile ranges (IQR) if distributed non-normally. Dichotomous and categorical variables were reported in frequencies and percentages. The comparison of the primary outcome and other PROMs were conducted by means of a Wilcoxon signed-rank test. Additionally, a sub-analysis for the improvement in the primary outcome were examined for patients who were classified as “responders” or “non-responders.” A patient was classified as a “responder” if a change of ≥ 2 out of 10 the NRS during walking from baseline to 2-year follow-up was achieved, as this was previously described as a “much better improvement in pain,” and no conversion to surgery had occurred.^13^ Moreover, the baseline characteristics for responders and non-responders were comparing with a Mann-Whitney *U* test for continues variables and Fishers’ exact test for dichotomous variables. To test the between group difference within each follow-up point (i.e., baseline, 6 months, 1 year, and 2 years) an adjusted *P*-value was used (0.05/4 = 0.0125). Lastly, a sub-analysis was conducted by comparing the change in PROMs from baseline to 2-year follow-up of patients with a solitary OLTP compared to bipolar (i.e., OLTP with coexisting OLT) lesions with a rank-sum test. An inter-observer intra-observer (with minimum 2-month interval) reliability assessment for the lesion size measurements was performed with a 2-way mixed effects interclass correlation coefficient (ICC) model with absolute agreement. The ICC analysis was interpreted by the following cut-offs: 0.41–0.60 fair agreement, 0.61–0.80 moderate agreement, and 0.81–1.00 substantial agreement.

## Results

**Figure 1.**
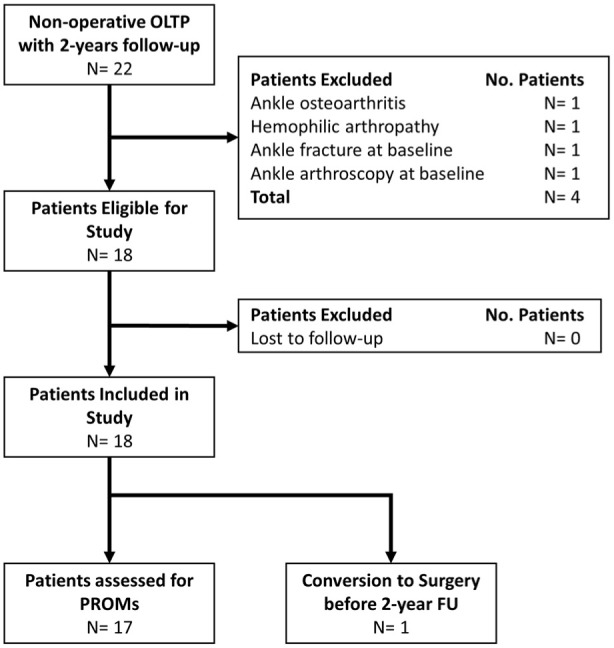
Flowchart of patient selection.

**Figure 2.**
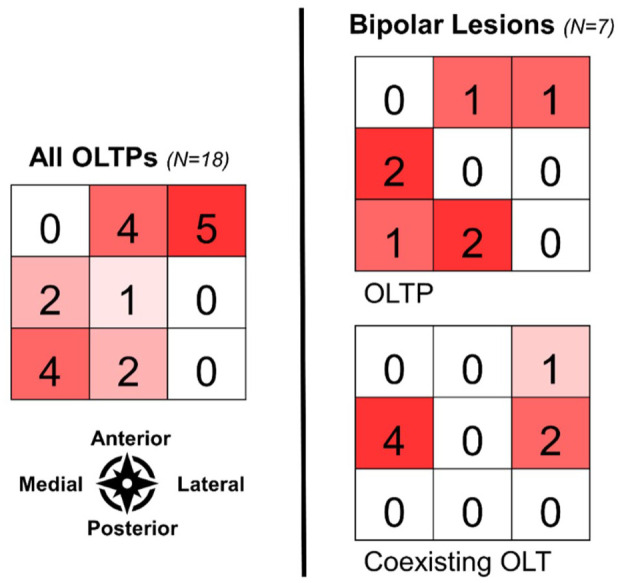
Heatmap of OLTP localization and bipolar OLTP with a coexisting OLT.

During the study period, 22 patients were assessed for eligibility, of which 18 were included. An overview of the patient selection process is available in **Figure 1**. An overview of the baseline patient characteristics is available in **Table 1**, the treatment characteristics in **Table 2**, and the lesion characteristics in **Table 3**. Lesion localization is depicted in **Figure 2**.

**Table 1.** Baseline Patient Characteristics.

Variable	Value	% Reported
Sex, *N male (%)*	12 (67%)	100
Age, *Years (SD)*	35.8 ± 11.4	100
BMI *(SD)*	24.3 ± 3.6	100
Traumatic Etiology, *N (%)*		95
- No trauma	4 (24%)	
- Ankle fracture	7 (41%)	
- Inversion/eversion or distortion	5 (29%)	
- Other	1 (6%)	
Sports Participation, *N* (%)	14 (78%)	100
* - Detailed, N (% of participating)*		
- Fitness	2 (14%)	
- Cycling or mountain biking	4 (29%)	
- Walking/hiking	2 (14%)	
- Racket sport (tennis, padel, badminton)	2 (14%)	
- MMA/kickboxing	2 (14%)	
- Other	2 (14%)	
Prior Ankle Surgery^a^, *N (%)*		100
- Ankles	9 (50%)	
- Total no. prior procedures	20	
*Detailed, N (% of total no. prior surgeries)*		
- External fixation ankle fracture	2 (10%)	
- ORIF ankle fracture	4 (20%)	
- Hardware removal	5 (25%)	
- Ankle arthroscopy		
○ BMS OLTP	3 (15%)	
○ BMS OLT	1 (5%)	
○ Diagnostic arthroscopy	2 (10%)	
○ Removal bony impingement	1 (5%)	
- OATS OLT (open)	1 (5%)	
- Malunion correction calcaneus	1 (5%)	

*N* = number of; BMI = body mass index; BMS = bone marrow stimulation; OLTP = osteochondral lesion of the tibial plafond; OATS = osteochondral autograft transplantation system; OLT = osteochondral lesion of the talus; SD = standard deviation.

a One ankle could have had more than one prior surgery for the OLTP.

**Table 2.** Treatment Characteristics.

Variable	Value	% Reported
Number of Non-Operative Treatments^a^, Mean (SD)	2.3 ± 1.1	100
*Specified per Treatment, N (% of total)*		
- Physical Therapy	13 (31%)	
- Supervised Neglect	5 (12%)	
- Injection hyaluronic acid	9 (21%)	
- Weight-loss advise	2 (5%)	
- Insole	9 (21%)	
- Brace or immobilizer		
○ Brace during exercise	3 (7%)	
○ 6 weeks of NWB cast	1 (3%)	
Concomitant diagnosis^a^, *N (%)*		
- Ankles	10 (56%)	
- Total no. concomitant diagnosis	13	
*Specified (% by no. concomitant diagnosis)*		
- Anterior bony impingement	6 (45%)	
- Anterior soft-tissue impingement	1 (8%)	
- Sinus tarsi syndrome	2 (15%)	
- Hardware irritation	1 (8%)	
- Lateral ankle instability	1 (8%)	
- Posterior tibial tendon tendinitis	1 (8%)	
- Malunion distal fibula	1 (8%)	

*N* = number of; SD = standard deviation; NWB = non-weightbearing.

a One ankle could have had more than one concomitant diagnosis, and more than one non-operative treatment for the OLTP.

**Table 3.** Baseline Lesion Characteristics.

Variable	Value	% Reported
Primary OLTP lesion, *N (%)*	16 (89%)	100
Coexisting talar lesion, *N (%)*	7 (39%)	100
Presence cyst, *N (%)*	13 (72%)	100
Lesion Morphology, *N (%)*		100
- Cystic	12 (67%)	
- Crater	5 (28%)	
- Fragment	1 (5%)	
OLTP Lesion Size, *median (IQR)*^a^		100
- Anterior-Posterior	10 (7 – 15)	
- Medial-Lateral	10 (6 – 14)	
- Depth	7.5 (5 – 10)	
Coexisting OLT Size, *median (IQR)*^a^		100
- Anterior-Posterior	5 (3 – 15)	
- Medial-Lateral	5 (4 – 12)	
- Depth	4 (3 – 9)	

*N* = number of; OLTP = osteochondral lesion of the tibial plafond; mm = millimeters; OLT = osteochondral lesion of the talus.

a Data not distributed normally, therefore represented with as medians with interquartile ranges (IQR).

### Patient-Reported Outcomes

**Figure 3.**
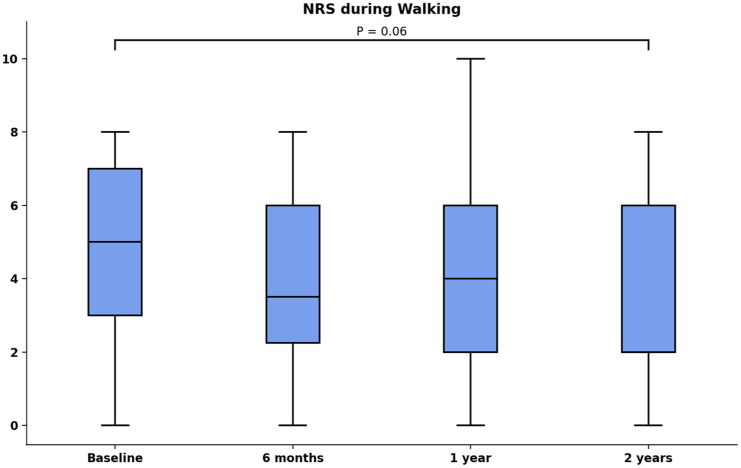
NRS during walking over time, of note, the outcome was not available for 3 patients at 6-month follow-up.

The primary outcome, the NRS during walking did not significantly change from median of 5 (IQR: 3–7) at baseline to 2 (IQR: 1–6) at 2-year follow-up, *P* = 0.06. The primary outcome did not significantly change from 6 months to 1-year or 2-year follow-up (**Figure 3**). All NRS subscales, the FAOS, and SF-36 subscales did not significantly change (**Table 4**).

**Table 4.** Patient-Reported Outcome Measures at Baseline and 2-Year Follow-Up.

Outcome	Baseline	2-Year Follow-Up	*P*-Value
**NRS pain during**, *Median (IQR)*			
Walking	5 (3 – 7)	2 (2 – 6)	0.1
Rest	2 (1 – 4)	1 (0 – 3)	0.2
Running	7 (4.5 – 8.5) *N = 16*	5 (3 – 9) *N = 15*	0.2
Stair Climbing	5 (4 – 7) *N = 16*	2 (1.5 – 6.5) *N = 16*	0.1
**FAOS**, *Median (IQR)*		*N* = 14	
Symptoms	64 (50 – 71)	59 (50 – 75)	0.7
Pain	64 (53 – 72)	67 (44 – 89)	0.7
ADL	98 (97 – 98)	98 (97 – 100)	0.8
Sport	40 (15 – 50)	43 (10 – 95)	0.1
QoL	31 (19 – 38)	41 (13 – 56)	0.4
**SF-36**, *Median (IQR)*		*N* = 13	
PCS	38.0 (35.7 – 42.0)	41.0 (37.5 – 44.1)	0.6
MCS	32.0 (30.5 – 36.8)	31.3 (29.2 – 39.8)	0.6

NRS = numeric rating scale; FAOS = foot and ankle outcome score; IQR = interquartile range; ADL = activities of daily living; QoL = quality of life; SF-36 = short-form 36; PCS = physical component scale; MCS = mental component scale.

**Figure 4.**
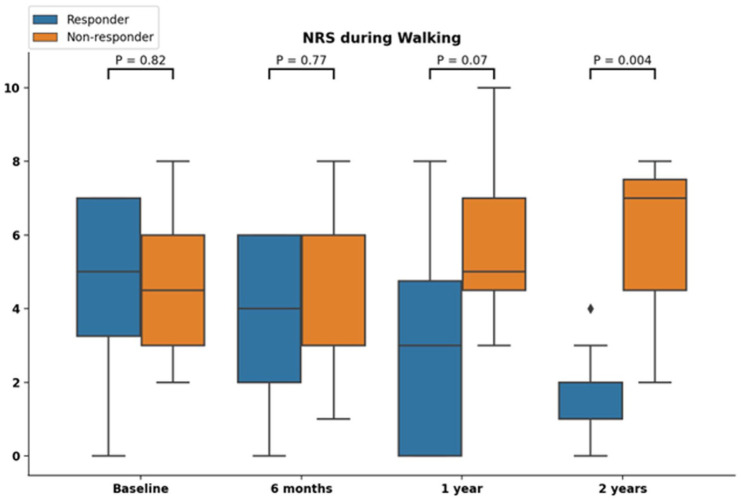
NRS during walking categorized according to “responder” or “non-responder” to treatment.

When examining the sub-analysis for responders compared to non-responders we observed that 10 (55%) patients were classified as responders. Responders had a significantly lower NRS for pain during walking (median 2 [IQR: 1–2]) at final follow-up compared to non-responders (median 7 [IQR: 3–8]) *P* < 0.01), and a different clinical course (**Figure 4**). Additionally, we observed there were no significant differences in baseline characteristics (Appendix A). Moreover, there were no significant differences in change of PROMs from baseline to follow-up when comparing solitary OLTP to bipolar lesions (Appendix B).

### Radiological Outcomes

At median 2 years (IQR: 1.5–2) follow-up CT-scan examination was available for 13 patients. Median lesion volume did not significantly change at final follow-up (219 [IQR: 75–552] mm^3^) compared to baseline (226 [IQR: 79–890] mm^3^), *P* = 0.2. In 4 (31%) cases a stable lesion was observed, in 6 (46%) cases lesion healing was observed, and in 3 (23%) cases deterioration was observed. A qualitative description of lesion healing is provided in Appendix C. The ICC measurements all showed substantial (>0.81) agreement and are available in Appendix D.

### Sports and Work Outcomes

Of the patients who participated in sports at baseline 93% (13/14) returned to any level of sports. Of these, 54% (7/13, 1 case unknown) returned to preinjury level of sports. Of the 4 patients who did not participate in sports at baseline, 1 (25%) patient started sporting activities (fitness).

At baseline, 16 patients worked and 2 patients were unemployed. Of the employed patients, 15 (94%) returned to work or remained working. Of these, 14 patients remained at their preinjury occupation and 1 patient returned to a part-time non-physically demanding occupation. A detailed overview of the return to sports and work outcomes per patient is provided in Appendix E.

### Treatment Failure and Adverse Outcomes

In this cohort, 1 (6%) patient underwent a surgical intervention of the ankle within the study period at 7 months after initiating non-operative treatment due to ongoing symptoms. The patient underwent an osteotomy and filling with autologous bone grafting procedure. At 2-year follow-up, this patient achieved satisfactory pain levels and functional outcomes in activities of daily living and returned to sports (tennis) at any level. No adverse events in any of the included patients were observed during the study period.

## Discussion

The principle finding of this study was that non-operative management for OLTP results in minor improvements of patient-reported pain and functional outcomes up to 2-year follow-up. In this cohort 1 patient (6%) required conversion to surgical treatment. Radiologically, lesion size and filling were found to remain stable at CT follow-up. Moreover, on average 9 out of 10 patients were able to participate in sport and could return to, or remain at, their preinjury work activities.

### Patient-Reported Outcomes

When comparing the results of the present study to the literature on non-operative OLT management it can be stated that these largely concur, in the sense that non-operative management shows limited success.^7,14^ Although the OLT literature on non-operative management is larger than compared to OLTPs it should be stated that it, too, is limited as mainly it consists of retrospective studies which may overestimate treatment effects due to selection bias (e.g., not including patients which converted to surgery in their PROMs assessment).^7^ Concerning the observed patient-reported outcomes in the present study, the authors note that these do not point to a substantial *clinically relevant* improvement and that minor improvements in pain and functional outcomes were obtained. This should be considered when counseling patients with an OLTP, as from the present results it cannot reasonably be expected that most patients achieve substantial improvements in clinical outcomes. It is therefore importance to further study which patients with an OLTP may benefit from non-operative management. We observed that 55% of cases could be considered a “responder” (i.e., improvement of 2 points NRS during walking) to non-operative management. Such patients showed a different clinical pathway in terms of pain outcomes compared to “non-responders, with continued improvement up to 2-year follow-up. We could not identify any differences in baseline characteristics between responders and non-responders. It should be noted, however, that this sub-analysis contains a low number of patients and that a “response,” as defined in this study, should not necessarily mean treatment success for a patient. In clinical practice it is therefore critical to assess the effect of non-operative management on an individualized level. Moreover, we assessed whether if a concomitant OLTP would affect clinical outcomes in a sub-analysis, which is hypothesized in BMS for OLTP with a concomitant OLT.^15,16^ This analysis found no differences but was likely underpowered.

Another point to consider from the findings of this study is that patient-reported outcomes and radiological outcomes on a group level did not deteriorate, meaning that it can be considered a safe treatment alternative, as was previously observed in OLT.^17,18^ When counseling patients for the treatment of an OLTP the pros and cons of non-operative management versus surgery should be weighed. On the one hand, non-operative management could reasonably be used as a first-line treatment for OLTP as it appears safe, possibly avoiding the need for surgical intervention, as is the clinical recommendation in OLT.^7,8^ Moreover, the effects of non-operative management could be assessed at 6-month follow-up due to no significant improvements being observed hereafter. On the other hand, it could be recommended that surgical intervention can be considered at 6 months of treatment if complaints deteriorate or are at an unacceptable level for patients. It should be stated, however, that the reliability of surgical treatment results remains limited due to the current limited low level of evidence literature.^5,6^

### Radiological Examination

We observed no significant change in lesion volume, and 77% of lesions showed either no change or a healing tendency of the OLTP at follow-up CT-evaluation. A systematic review by Buck *et al*,^7^ assessing non-operative management for OLT among 30 studies evaluated lesion deterioration on CT for 131 ankles. The authors observed deterioration of the OLT in 11% of patients, while the lesion was found unchanged in 76% on CT and 83% on magnetic resonance imaging (MRI).^7^ Although not wholly comparable to the present study as these findings solely concern OLT, one could state that these findings concur with the present study. This suggests that lesions remain stable over time, showing no clear adverse effects from non-operative treatment on lesion size and filling based on these data. Moreover, the authors advocate the use of follow-up CT-scan examinations in symptomatic OLTP cases where non-operative management is started.^7^ It could be argued that this is required in all cases as, up-to this point in time, little is known about the natural history of OLTP. Routine CT-evaluation can assist in clinical decision making by early detection of clinically significant lesion change (e.g., size increase or cyst formation) and assists with informing patients as well as expectation management. However, there is no evidence on the timing and cost-effectiveness of routine CT-examination in OLTP and its use should, therefore, be considered in a case-based and shared decision-making manner.

### Sport and Work Outcomes

Sport outcomes reported for the operative management of OLTP range from 63% to 83% return to any level of sports.^5^ The present literature on sports outcomes for OLTP concerns a low number of patients, among heterogenous treatment groups, and lacks clear definitions of return to sport, such as postulated by Ardern *et al*.^10^ Moreover, in the present study we observed that several patients did not stop with their sporting activities during treatment, for example as would be common following surgery. This makes defining clear end-points difficult, and we thus focused on sports-participation based on any-level or preinjury level of sports.^10^

With regard to the work outcomes, it was observed that 94% of patients returned to work or remained at their occupation. Moreover, of those patients who remained at their occupation 4 patients initiated some kind of work modification, which were primarily focused on reducing weightbearing activities. Future studies should assess the impact of OLTP on work outcomes as these are important outcome measures for patients.

### Conversion to Surgery and Adverse Outcomes

A notable finding was that 1 (6%) patient converted to a surgical intervention within the study period. This is comparatively low to what is reported in OLT literature (46%, evaluated in 400 ankles).^7^ This could be explained by the tertiary academic referral setting of our institution and shared decision-making process at initial clinical consultation. As a tertiary referral center for ankle cartilage lesions most patients had already undertaken a period of non-operative management before presenting at our institution and could therefore have been more inclined to undergo surgery. As such, it could be hypothesized that the patients in this cohort were less inclined to convert to a surgical intervention as they initially chose to commence non-operative treatment. The present cohort may, therefore, be subject to selection bias. On the other hand, patient expectations, treatment goals, and pain coping may have changed during the study period, which could influence the decision to convert to surgery. Such factors were not measured in the questionnaires. Implementing a standard period of up to 6 months of non-operative management in initially diagnosed OLTP cases and prospectively examining these could elucidate the conversion rate of non-operative management in the general OLTP population.

### Strengths and Limitations

The strengths of this study are its prospective design with 2-year follow-up, clinical relevance through its novelty with regard to non-operative treatment for OLTP, sample size calculation, and use of multiple assessors for the radiological outcomes. Moreover, there was a 100% follow-up rate for the primary outcome measure, and patients were assessed through a wide spectrum of outcomes.

The present study is not without its limitations. First, patients underwent a heterogenous number of treatments within non-operative management and timing of these treatments. The effect of these specific sub-treatments could not be assessed as there was insufficient power, which should further be investigated in larger cohort studies. Second, patients presented with a number of concomitant diagnoses besides the OLTP, including concomitant OLT. The authors excluded all cases of asymptomatic OLTP, but it could be argued that such concomitant diagnoses were in part responsible for the complaints and treated during the 2-year study period and as such the present study did not wholly assess the effect of non-operative management on OLTP alone. Finally, the present study did not have a complete follow-up of 1-year CT scans and a number of patients did not fully complete the questionnaires resulting in a number of incomplete secondary patient-reported outcome measures which could have introduced bias.

## Conclusion

Non-operative management for OLTP resulted in minor improvement of patient-reported pain and functional outcomes up to 2-year follow-up. In this cohort, 1 patient (6%) required conversion to surgical treatment. Radiologically, lesion size and filling were found to remain stable at CT follow-up. Moreover, on average 9 out of 10 patients were able to participate in sport and could return to, or remain at, their preinjury work activities.

## Supplemental Material

sj-docx-1-car-10.1177_19476035251376180 – Supplemental material for Non-Operative Management for Osteochondral Lesions of the Tibial Plafond Results in Minor Improvements of Patient-Reported Outcomes: A 2-Year Prospective Follow-Up Study Supplemental material, sj-docx-1-car-10.1177_19476035251376180 for Non-Operative Management for Osteochondral Lesions of the Tibial Plafond Results in Minor Improvements of Patient-Reported Outcomes: A 2-Year Prospective Follow-Up Study by Quinten G.H. Rikken, Jari Dahmen, Sjoerd A.S. Stufkens and Gino M.M.J. Kerkhoffs in CARTILAGE

## Appendices

**Appendix A.** Sub-Analysis of Baseline Characteristics of Responders and Non-Responders to Non-Operative Management.

	Responders *N* = 10	Non-Responders *N* = 8	*P*-Value
**Patient Characteristics**			
Sex, *N male (%)*	7 (70%)	5 (63%)	0.9
Age, *Years (median, IQR)*	38.5 (25 – 40)	32.5 (25 – 42.5)	0.9
BMI *(median, IQR)*	23.8 (21.8 – 27.7)	24.1 (21.6 – 25.5)	0.8
**Treatment Characteristics**			
Number of Non-Operative Treatments^a^, *median, (IQR)*	2.5 (1 – 3)	2 (2 -3)	0.9
*Specified per Treatment, N (% of total)*			
- Physical Therapy	8 (33%)	5 (28%)	
- Supervised Neglect	3 (13%)	3 (16%)	
- Injection hyaluronic acid	4 (17%)	5 (28%)	
- Weight-loss advise	2 (8%)	0 (0%)	
- Insole	4 (17%)	4 (22%)	
- Brace or immobilizer			
○ Brace during exercise	2 (8%)	1 (6%)	
○ 6 weeks of NWB cast	1 (4%	0 (0%)	
**Lesion Characteristics**			
Primary OLTP lesion, *N (%)*	9 (90%)	7 (88%)	0.9
Coexisting talar lesion, *N (%)*	4 (40%)	3 (38%)	0.9
Presence cyst, *N (%)*	7 (70%)	6 (75%)	0.9
OLTP Lesion Size, *median (IQR)*			
- Anterior-Posterior	10 (9 – 14)	7 (4.5 – 17.5)	0.4
- Medial-Lateral	10 (8 – 13)	10.5 (6 – 17)	0.8
- Depth	7.5 (6 – 10)	7 (4.5 – 14)	0.8

a One ankle could have had more than one prior surgery for the OLTP.

**Appendix B.** Change in PROMs From Baseline to 2-Year Follow-Up Compared for Solitary OLTP to Bipolar Lesions.

Median Change (Improvement)	Solitary OLTP *N* = 11	Bipolar Lesions *N* = 6^a^	*P*-Value
**NRS pain during**, *Median (IQR)*			
Walking	1 (0 – 2)	3.5 (3 – 5)	0.3
Rest	0 (0 – 2)	1 (- 2 – 2)	0.9
Running	1 (-1 – 2)	1.5 (0.5 – 4.5) *N = 4*	0.6
Stair Climbing	1.5 (-1 – 3)	2 (1 – 3)	0.9
**FAOS**, *Median (IQR)*	*N = 10*	*N = 4*	
Symptoms	0 (-11 – 11)	7 (0 – 14)	0.2
Pain	0 (-8 – 6)	10 (-3 – 24)	0.3
ADL	0 (0 – 2)	-1 (-45 – 2)	0.6
Sport	8 (0 – 35)	10 (-2.5 – 35)	0.9
QoL	3 (-6 – 19)	0 (-3 – 28)	0.9
**SF-36**, *Median (IQR)*	*N = 9*	*N = 4*	
PCS	0 (-1 – 3)	2 (- 2 – 10)	0.8
MCS	0.5 (-1 – 3)	- 4 (-5 – 0)	0.5

a Of note: 1 of the bipolar patients was not included as the patient underwent a re-operation before the 2-year follow-up time-point.

**Appendix C.** Per-Case Description of Radiological Follow-Up Outcomes.

	Baseline					Follow-up	
Patient	Primary lesion	Location	Size (AP x ML x depth), mm	Cyst	Coexisting OTL	Size (AP x ML x depth), mm	Healing/lesion filling description
1	Yes	Zone 2	8 x 3 x 3	Yes	No	8 x 4 x 4	Stable lesion, no markable change in filling or size
3	Yes	Zone 3	10 x 20 x 22	Yes	No	9 x 16 x 20	Moderate lesion filling of cysts
6	Yes	Zone 3	4 x 5 x 4	Yes	No	5 x 4 x 4	Stable lesion, no markable change in filling or size
7	Yes	Zone 7	4 x 6 x 5	Yes	No	5 x 7 x 7	Moderate increase cyst formation and lesion size
8	Yes	Zone 8	14 x 13 x 12	Yes	Yes	14 x 12 x10	Moderate lesion filling of cysts
9	Yes	Zone 8	7 x 4 x 6	Yes	Yes	6 x 6 x 4	Moderate lesion filling of cysts
10	Yes	Zone 7	17 x 10 x 10	No	Yes	11 x 7 x 9	Considerable lesion filling of crater
11	Yes	Zone 7	9 x 9 x 6	No	No	8 x 8 x 5	Stable lesion, no markable change in filling or size
14	Yes	Zone 3	20 x 22 x 45	Yes	No	19 x 18 x 39	Moderate lesion filling of cysts
15	No	Zone 2	8 x 13 x 3	No	Yes	11 x 9 x 7	Moderate increase cyst formation
16	Yes	Zone 4	6 x 8 x 9	Yes	Yes	7 x 6 x 10	Stable lesion, no markable change in filling or size
17	Yes	Zone 2	10 x 12 x 7	Yes	No	11 x 12 x 8	Moderate increase cyst formation
18	Yes	Zone 7	5 x 6 x 5	No	No	3 x 3 x 2	Considerable lesion filling of crater

**Appendix D.** Inter-Observer Reliability Measurements for Lesion Size Measurements.

	AP	ML	Depth
Inter-observer reliability	0.97 (0.86 – 0.99)	0.97 (0.45 – 0.99)	0.99 (0.84 – 0.99)
Intra-observer reliability (rater 1)	0.97 (0.88 – 0.99)	0.99 (0.95 – 0.99)	0.98 (0.94 – 0.99)

**Appendix E.** Overview of Sports and Work Outcomes Per Case.

	Baseline			2-Year Follow-up					
	Sport Outcomes	Work Outcomes		Sport Outcomes			Work Outcomes		
Patient	Sport and level	Occupation	Hours of work per week	Return to any level of sport	Sport and level	Return to preinjury level of sport	Occupation, return to work	Hours of work per week	Work modifications
1	Running, recreational level	Hairdresser (physical labor)	Full-time (hours unknown	No	None	No	As baseline, yes	32	Part-time office work to reduce hours standing
2	Mountain biking, recreational level	Office job (sitting)	36	Yes	As baseline	Yes	As baseline, yes	36	None
3	Fitness, recreational level	Office job (sitting)	36	Yes	Tennis, Ice skating, recreational level	Yes	As baseline, yes	36	None
4	Kitesurfing, recreational level	Office job (sitting)	Part-time (hours unknown	Yes	As baseline	Yes	As baseline, yes	40	None
5	Mountain biking, recreational level	Office job (sitting)	40	Yes	As baseline	Unknown	As baseline, yes	40	None
6	Kickboxing recreational level, fitness, competitive level	None	N/a	Kickboxing: No, Fitness: Yes	Fitness, competitive level	Fitness: Yes	None	N/a	N/a
7	None	Gardener (physical labor)	40	N/a	None	N/a	As baseline, yes	40	Reducing standing time
8	None	Warehouse worker (physical labor)	32	N/a	None	N/a	Returned to non-physical charity work, No	12	Stopped work
9	Fitness, recreational level Mixed martial arts, recreational level	Steel worker (physical labor)	Unknown	Fitness: Yes, Mixed martial arts: No	Fitness, recreational level	No	Office job (sitting)	24	Re-educated to non-physical occupation
10	Walking, recreational level	Office job (sitting)	36	Yes	Walking, recreational level Padel, recreational level	Yes	As baseline, yes	36	None
11	None	Office job (sitting)	Full-time (hours unknown	N/a	None	N/a	As baseline, yes	Full-time (hours unknown	Unknown
12	Walking, recreational level	Office job (sitting)	Full-time (hours unknown	Yes	Walking, recreational level Padel, recreational level	No	As baseline, yes	40	Reducing standing time
13 (conversion to surgery)	Tennis, recreational level Swimming, recreational level	Office job (sitting)	40	Yes	Tennis, recreational level Swimming, recreational level Padel, recreational level	Yes	As baseline, yes	40	None
14	Cycling, recreational level	Truck driver	40	Yes	As baseline	No	As baseline, yes	40	Reducing physical activities during work
15	None	None	N/a	N/a	None	N/a	Cleaning	40	Unknown
16	Fitness, recreational level	Office job (sitting	Full-time (hours unknown	Yes	As baseline	No	As baseline, yes	Full-time (hours unknown	None
17	Mountain biking, recreational level	Office job (sitting	50	Yes	As baseline	No	As baseline, yes	50	None
18	Tennis, recreational level	Office job (sitting	50	Yes	Fitness, recreational level	Yes	As baseline, yes	40	None
