# Supplementary material for: Non-Operative Management for Osteochondral Lesions of the Tibial Plafond Results in Minor Improvements of Patient-Reported Outcomes: A 2-Year Prospective Follow-Up Study
Source: Cartilage. 2025 Sep 27:19476035251376180. Online ahead of print. doi: 10.1177/19476035251376180 (PMC12476373; doi:10.1177/19476035251376180)
Supplement: sj-docx-1-car-10.1177_19476035251376180 – Supplemental material for Non-Operative Management for Osteochondral Lesions of the Tibial Plafond Results in Minor Improvements of Patient-Reported Outcomes: A 2-Year Prospective Follow-Up Study [file sj-docx-1-car-10.1177_19476035251376180.docx]

| **Inclusion Criteria** | **Exclusion Criteria** |
| --- | --- |
| Symptomatic osteochondral lesion of the tibial plafond with 2-year follow-up as assessed through clinical evaluation (history and physical examination) | End-stage ankle osteoarthritis (Kellgren-Lawrence grade ≥3) at baseline |
| AND | Concomitant ankle fracture at baseline |
| Lesion confirmed by computed tomography (CT) scan | Patients unwilling or unable to participate |
|  | Systemic disease affecting the ankle; including rheumatoid arthritis and hemophilic arthropathy |
|  | Patients lost-to-follow-up |
|  | Patients undergoing ankle arthroscopy at baseline |

**Supplemental Table 1: Inclusion- and Exclusion Criteria**
